# Pandemic panic and anxiety in developing countries. Embracing One Health offers practical strategies in management of COVID-19 for Africa

**DOI:** 10.11604/pamj.2020.35.3.22637

**Published:** 2020-04-14

**Authors:** Keneth Iceland Kasozi, Regan Mujinya, Paul Bogere, Justine Ekou, Gerald Zirintunda, Salaviriuse Ahimbisibwe, Kevin Matama, Herbert Izo Ninsiima, Isaac Echoru, Emmanuel Tiyo Ayikobua, Godfrey Ssimbwa, Simon Peter Musinguzi, Robert Muyinda, Fred Ssempijja, Henry Matovu, Ewan MacLeod, Neil Euan Anderson, Susan Christina Welburn

**Affiliations:** 1 Infection Medicine, Deanery of Biomedical Sciences, College of Medicine and Veterinary Medicine, University of Edinburgh, United Kingdom; 2 Faculty of Biomedical Sciences, Kampala International University Western Campus, Box 71 Bushenyi, Uganda; 3 Department of Animal Production and Management, Faculty of Agriculture and Animal Sciences, Busitema University, Box 236 Tororo, Uganda; 4 The Royal (Dick) School of Veterinary Studies and the Roslin Institute, University of Edinburgh, Roslin, EH25 9RG, United Kingdom; 5 Department of clinical pharmacy and Pharmacy practice, School of Pharmacy, Kampala International University Western Campus, Box 71 Bushenyi, Uganda; 6 Department of Physiology, School of Medicine, Kabale University, Kabale, Uganda; 7 Department of Anatomy, School of Medicine, Kabale University, Kabale, Uganda; 8 Department of Physiology, School of Health Sciences, Busitema University; 9 Department of Physiology, School of Health Sciences, Soroti University, Soroti, Uganda; 10 Department of Microbiology and Immunology, School of Medicine, Kabale University, Kabale, Uganda; 11 Zhejiang University-University of Edinburgh Institute, Zhejiang University School of Medicine, International Campus, Zhejiang University, 718 East Haizhou Road, Haining, Zheijang 314400, Peoples Republic of China

**Keywords:** Coronaviruses, COVID-19, Africa, pandemic, one health

## To the editors of Pan African Medical Journal

The coronavirus, COVID-19 outbreak has now affected over 60% of African countries in less than two months [1], gaining a foothold through major economic and transport hubs on the African continent including Egypt, Algeria, Nigeria, South Africa and Kenya. Travel restrictions imposed against citizens from countries with major outbreaks including China, USA and those in Europe were too late [2,3]. African Union member states as of early April 2020 are reporting 6,470 cases and 241 deaths from COVID-19 reporting growth as “close to exponential”. Africa Centers for Disease Control and Prevention acknowledges the virus is an existential threat to African countries and that with local transmission now underway many would pass the 10,000-infection mark by the end of April [4]. While the impact of wearing of face masks for control of COVID-19 remains controversial, it is inarguable that respiratory transmission needs to be prevented. Currently, there is a global shortage of masks and personal protective equipment (PPE) and distribution is being rationed in developed countries to retain this for workers in the health system [5], showing that developing countries in Africa are bound to suffer more should the pandemic be mismanaged at these early stages. In addition, health systems in developing countries, already crippled from years of underinvestment will be compromised unless practical and realistic prevention strategies are put in place. China, Italy, France, UK and USA, all with sophisticated health systems, have found COVID-19 challenging. Infection is increasing across the African subcontinent and health systems will struggle as the pandemic sweeps into and across Africa.

For management of pandemics a One Health approach can be efficient and cost-effective. This is because One Health approaches comprise cross-disciplinary collaborations and interdisciplinary interventions involving veterinary, medical, ecological and public health authorities and its importance is undisputed in the control of complex zoonotic diseases [6]. One Health was fueled by the global anxiety during the HPAI H5N1 pandemic fear. The introduction of the One Health initiative provided international agencies i.e. the Food and Agricultural Organization (FAO), Organization for Animal Health (OIE), World Health Organization (WHO) and the World Bank, with a vehicle for interinstitutional and interdisciplinary collaboration to address the threat of emerging zoonotic diseases, and it enabled these international agencies and national authorities to come to the table as equal partners in the search for solutions [7]. For management of Rift Valley fever (a highly infectious zoonotic disease in Africa involving animals and humans) poor strategies for disease control resulted in 23 avoidable deaths amongst Africans from a lack of co-ordination [8] demonstrating a live situation in which One Health adoption was delayed. Furthermore, human transmission of COVID-19 to pets has been reported and no evidence of animal to human transmission [9].

Globally, national governments have largely followed the general World Health Organization (WHO) guidelines on prevention against COVID-19. Preventative measures include hand washing with soap, use of alcohol based sanitizers, banning of social events, and national lockdowns of non-essential business activities. While these preventive models have worked, or appear to be working to slow and lower the peak of infections (and deaths) that are expected, in Africa, resistance to these measures is building up in grassroots communities. This has been in part due to the socio-economic challenges in slum communities, common in many low and middle-income countries; a chronic lack of any savings (money) to buy food and the threat of hunger while staying at home [10]. Governments are required to adopt and deploy robust but flexible strategies to secure the economy and protect their citizens. Excessive force by the police and armed forces has already led to alleged serious human rights violations in South Africa and Kenya [10]. COVID-19 control strategies have led to panic and anxiety in countries where the majority of citizens live below the poverty line, and lack the ability to fully comply with WHO recommendations. Many countries in Africa are already facing challenges arising from severe malnutrition, crippled healthcare systems and the absence of a robust national disease task force and health leadership [11]. The WHO has trained field epidemiologists from Democratic Republic of Congo, Kenya, Zambia, South Africa, Uganda, Zimbabwe and Mozambique on pandemic One Health management [12] but there has been little time to mobilize training into action.

In Africa, much focus has been put on the Ministry of Health, and in some countries, massive recruitment of additional health staff is in progress. While noble, these rapid recruitments communicate panic and increase anxiety and stress in a population fighting an invisible enemy. There is as yet no local name in any African dialect for COVID-19. In several developing countries in Africa, the Ministry of Local Government or Public Service has administrative units at every district or county or provincial level, however, these have not been fully integrated effectively into the National COVID-19 Control Task Force, with current approaches being controlled by the Ministry of Health and the Office of the President. Implementing the WHO advice “test, test, test” for COVID-19, to manage transmission will require surveillance and testing of large numbers of the population, many of whom will be hard to access. Establishment of a coordinated disease control task force comprising of essential staff drawn from many different sectors i.e. medical officers, veterinarians, community psychologists, local leaders will improve the efficiency and effectiveness of the management of COVID-19. Working with the Animal Health Ministries can facilitate access to rural communities. A range of support professionals under the Ministry of Public service or Ministry of Local Government, are available and able to work in partnership with public health professionals and medical staff at a time when their medical counterparts are overstretched. Funding, to mobilize all public servants to contribute to management of COVID-19 is needed at this time.

Detection of COVID-19 patients relies on symptomology which is highly variable. The presence of pre-symptomatic and asymptomatic individuals is a major challenge for detecting infections and isolating individuals to prevent transmission. Many of the symptoms of COVD-19 are those associated with endemic tropical diseases that have persistently blighted the African subcontinent for example common colds or flu. Confirmatory diagnosis of infection with, or exposure to COVID-19, demands rapid diagnostic tests and laboratory diagnostic facilities. These will need to be sourced from the public and private sector, including universities and veterinary diagnostic providers. To reduce the risk of crippling the healthcare systems in Africa, it is essential and urgent that governments establish partnerships with private hospitals and universities (with the diagnostic capability) to manage demand. Only samples that provide inconclusive results would be sent to the national reference laboratory, reducing the burden on the human resource at the main center (e.g. the Uganda Virus Research Institute for Uganda or the Nigerian Centers for Disease Control). This would promote inter-institutional-national collaborations and pave a way for the establishment of an inter-ministerial national disease database in African countries to help control future pandemics on the continent. A reduction in the time spent transporting samples would also lead to a reduced exposure risk during sample transportation to the central national diagnostic laboratory. It is timely for Africa to ramp up its ambitions and commitments to One Heath. Now, more than ever, we can all appreciate that healthy people, healthy animals and healthy ecosystems are essential for heath today and to that of future generations. Investments to foresee and prevent zoonotic disease transmission are key to a sustainable and healthy future.

## Competing interests

All authors declare no competing interests.
